# Social support for youth physical activity: Importance of siblings, parents, friends and school support across a segmented school day

**DOI:** 10.1186/1479-5868-4-54

**Published:** 2007-11-08

**Authors:** Maea Hohepa, Robert Scragg, Grant Schofield, Gregory S Kolt, David Schaaf

**Affiliations:** 1 Centre for Physical Activity and Nutrition Research, Faculty of Health and Environmental Sciences, AUT University, New Zealand; 2 School of Population Health, University of Auckland, New Zealand; 3 School of Biomedical and Health Sciences, University of Western Sydney, Australia

## Abstract

**Background:**

Whilst evidence exists for the influence of encouragement on physical activity participation, the diversity of support sources and the type of physical activity examined previously is limited. This study examined the importance of perceived encouragement from parents, siblings/cousins, friends, and schools on participation levels across three time-specific activity opportunities that are available during a school day (after-school physical activities, lunchtime activity, and active transportation to and from school).

**Methods:**

A cross-sectional sample of 12–18 year old high school students (n = 3,471) were recruited from low SES schools within South Auckland, New Zealand and categorised as either Junior (Years 9–11) or Senior (Years 12 & 13) students. Participants reported their physical activity levels and quantity of encouragement received from their parent(s), friend(s), sibling(s)/cousin(s), and school to be active. For each physical activity variable participants were dichotomized as being either "active" or "less active". For each social support source, participants were grouped into either receiving "high" or "low" levels of support. Binary logistic regression analyzes were conducted to calculate odd ratios and 95% confidence intervals.

**Results:**

Low parental support (Juniors, OR: 0.47, 95% CI: 0.38–0.58; Seniors, OR: 0.41, 95% CI: 0.29–0.60) and low peer support (Juniors, OR: 0.61, 95% CI: 0.51–0.74; Seniors, OR: 0.49, 95% CI: 0.35–0.69) were associated with reduced odds of being regularly active after school. For lunchtime activity, low peer support (Juniors, OR: 0.39, 95% CI: 0.32–0.48; Seniors, OR: 0.41, 95% CI: 0.29–0.57) was associated with reduced odds of being categorized as active. While no variables were significantly related to active transportation among senior students, low peer support was associated with reduced odds of actively commuting for Junior students (OR: 0.78, 95% CI: 0.66–0.92). Irrespective of the activity examined, no significant difference was noted for students receiving high support from two parents than students reporting high support from their sole parent in a single parent family.

**Conclusion:**

The importance of encouragement from parents, siblings, friends, and schools on physical activity is dependant on the time-specific activity examined. It is clear that proximal social networks need to be considered during the development of physical activity promotion strategies.

## Background

The high school years is a period of life consistently associated with a subsidence in physical activity levels [1-5]. To slow down or reverse this trend, gaining a greater insight into the determinants of youth physical activity is required. Determinant-based frameworks of physical activity have been developed which focus on broad, multilevel, ecological health promotion approaches that work toward understanding the modifiable social and physical environmental determinants of physical activity [6,7]. Despite the recent upsurge and popularity in studies examining how the physical environment influences physical activity habits, further research into the relative importance of social support from various sources continues to be advocated [8].

Encouragement, role modelling, and logistical support are all examples of social support that have been positively associated with youth physical activity participation [8-18]. Among these elements, however, encouragement may have a longer lasting effect on behaviour change as not only does encouragement act directly on physical activity, but indirectly through its influence on self-efficacy [10,19], a key psychosocial variable repeatedly found to be associated with physical activity among young people [10,11,20-25].

Physical activity research, however, has been focused predominantly on two sources of encouragement – parents and friends – with little consideration of encouragement provided by siblings, extended family members (e.g., cousins), and schools. In New Zealand, living with extended family members is common among certain ethnic groups (e.g., Pacific Islanders) and therefore needs consideration [26]. When parental support has been examined, family type (e.g., no parents, two parent families, and single parent family) has rarely been considered. In 2001, 29% of families with dependent children in New Zealand were single parent families, placing New Zealand only behind America as having the second highest percentage of sole parent families among OCED countries [27]. Considering single parent families are disadvantaged economically and socially when compared to two-parent families [27] examining the influence of family type on youth physical activity levels is warranted. Furthermore, adolescence is a period of life characteristic of increasing independence from families and expanding social networks external to the family environment [28].

While prior studies support the encouragement – physical activity link, the studies have lacked diversity in the range of physical activities examined. Participation in vigorous or moderate-to-vigorous intensity physical activity has typically been examined with little attention directed towards activities undertaken at specific time periods or segments of a school day (e.g., after-school activity, lunchtime activity, before school through active transportation) in which young people can be active. Ecological models, which are holistic and consider both intraindividual and environmental correlates of the targeted behaviour (e.g., physical activity), posit that influencers most proximal to the target group will have the strongest effect on the desired behaviour [7]. Hence, the importance of support from parents, friends, siblings, and schools is likely to be dependent on the location, period of the day, and context of physical activity examined. Initiating this type of investigation, authors of a recent study of Norwegian youth aged 9 or 15 years old concluded that psychosocial correlates of physical activity appear to be location specific, but further examination is warranted to provide further insight. [29].

To overcome the identified gaps in literature, the aim of the current study was to simultaneously examine the importance of perceived encouragement from parents, siblings/cousins, friends, and schools on participation in after-school physical activities, lunchtime physical activity, and engagement in active transportation to and from school.

## Methods

### Data Collection

The OPIC (Obesity Prevention In Communities) project is an obesity prevention initiative focusing on high school aged students which is being conducted across four countries (Australia, New Zealand, Fiji, and Tonga). Within each country the project is being carried out within a predetermined suburb/neighborhood. The measures and data of this study represent a portion of the variables and data collected from schools participating in the New Zealand aspect of the OPIC project.

### Sample

Students were recruited from seven low SES (decile rating of 1 or 2) high schools located in South Auckland, New Zealand. The decile rating, which ranges from 1 (most deprived) to 10 (least deprived), indicates the extent to which the school draws its students from low socioeconomic communities. The response rate varied by school, from 25% up to 78% (school average = 58%). For all schools combined, the original sample surveyed in 2005 was 3,581 (response rate of 53% from 6,827 students) and from these 14 participants (0.4%) were excluded due to not meeting age criteria for inclusion (i.e., 12–18 years old, inclusively) with a further 96 participants (2.7%) excluded as a result of incomplete data. The final sample consisted of 3,471 participants (97% of the original sample) with a mean age of 14.8 ± 1.4 years, and a composition of 48% male, 72% junior students (Years 9–11) and a mix of different ethnicities (Pacific Island descent, 57%; Māori, 20%; European, 12%; Asian/Other, 11%). Written consent was gained from all students. For students aged below 16 years written parental consent was obtained for most students with a small number of parents providing consent orally over the phone. Consent was also gained from the principal of each school.

### Measures

Physical activity, perceived encouragement, and demographic variables were collected using an electronic (i.e., personal digital assistant, PDA) self-report questionnaire administered during a scheduled class time. A pilot study of the survey was conducted with four classes, one class at each year level (Year 9 through to Year 12), to examine comprehension level and survey completion time.

#### Demographic variables

The questionnaire requested information on age, gender, and ethnicity. For ethnicity, participants selected the main ethnic group they identified with from a list of New Zealand relevant ethnic groups. For the purpose of analyzes, students in Years 9, 10, and 11 were grouped as junior high school students, while senior high school students refers to those participants in Years 12 and 13.

#### Physical activity variables

The questionnaire contained three separate items to assess physical activity in the form of active transportation, activity during lunchtime and activity during the after-school time period. These items were directly replicated from the New Zealand Child Nutrition Survey (CNS) [30], a national survey of physical activity and nutrition among children. The New Zealand CNS survey was based on the Physical Activity Questionnaire for Children (PAQ-C), a questionnaire that has demonstrated acceptable reliability and validity [31-33]. Of the items that comprise the New Zealand CNS, questions that individually examined after-school activity and lunchtime activity were used in this study. Face/content validity of each question was assessed by the authors, and participant comprehension was tested during the piloting of the entire OPIC survey.

For after-school physical activity participants were asked to self-report the number of days (0–5 days) over the previous 5 school days they participated after school in sports, dance, cultural performances, or played games in which they were active. Based on their self-reported level of participation, participants were dichotomized into "active" (i.e., participated in after-school activities on at least 3 school days) or "less active" (i.e., participated in after-school physical activities on 2 or fewer days) groups.

In terms of lunchtime physical activity, participants were asked "over the last 5 school days, what did you do most of the time at lunchtime (apart from eating)". Participants chose one of the following three response options: 'mostly just sat down', 'mostly stood or walked around', or 'mostly played active games'. Based on their self-reported level of participation, participants were dichotomized into "active" (i.e., mostly played active games) or "less active" (i.e., mostly just sat down or mostly stood or walked around) groups.

For active transportation, each participant reported the number of trips he/she made by biking or walking to or from school over the previous 5 school days. Based on their self reported level of participation, participants were dichotomized into "active" (i.e., walked/biked to or from school for at least 5 trips in the previous school week) or "less active" (i.e., walked/biked to or from school for fewer than 5 trips in the previous school week) groups.

#### Perceived encouragement

Similar to items used in previous studies [10,34], perceived encouragement from the participant's mother, father, brothers/male cousins, sisters/female cousins, friends, and school was assessed individually using the following question format; "How much does your [*support source*] encourage you to be physically active or play sports". Participants responded using a 5-point response scale (a lot, some, a little, not at all, don't have/live with my [*support source*]).

Due to the potential of collinearity to occur between certain support sources, responses for maternal and paternal support were combined into a single independent variable referred to as 'parental encouragement' whilst responses for brother/male cousin and sister/female cousins were combined to form the independent variable of 'sibling/cousin encouragement'.

Based on their survey responses, participants were grouped into 'high' (i.e., reported receiving a lot of encouragement) and 'low' (i.e., reported receiving some to no encouragement) encouragement groups for each support source. The groups were then further divided according to their family structure (e.g., single parent family, two parent family, no parents) which was constructed from the participant's responses to maternal and paternal encouragement questions.

For parental encouragement, participants were classified as either receiving (1) high support from both parents in a two parent family, (2) high support from at least one parent from a two parent family, (3) high support from one parent within a single parent family, (4) low support from both parents within a two parent family, and (5) low support from their sole parent within a single parent family or does not live with his/her parents. In terms of sibling/cousin encouragement, participants were classified into one of three groups; high support from brothers/male cousins or sisters/female cousins, low (but not high) support from either brothers/male cousins or sisters/female cousins, and does not have siblings/cousins. For friend and school support, participants were grouped as either receiving high support or low support for each support source.

### Data analysis

Data were analyzed using Statistical Analysis System (SAS) version 9.1 (SAS Institute Inc., Cary, NC, USA), with corrections for any design effects arising from sampling students by class. Using binary logistic regression both univariate and multivariate analyzes were conducted to calculate odd ratios (crude and adjusted, respectively) and 95% confidence intervals. Both univariate and multivariate models were adjusted by sex and ethnicity.

Developing the multivariate model consisted of a two step process. First, the 'proc logistics' procedure in SAS was conducted to identify significant predictor variables based on mutual adjustment of all predictor variables while adjusting for sex and ethnicity also. All predictor variables with a p-value below 0.05 were identified and kept for the final multivariate model. As the variables had not been corrected for cluster sampling during this process, the final model was then tested through the 'Proc surveylog' procedure to calculate adjusted odds ratios (OR's) and 95% confidence intervals (95% CI) that were adjusted by sex and ethnicity and corrected for cluster sampling.

## Results

### After-school physical activity

Univariate analyzes (Table 1) showed that encouragement from all sources (with the exception of school support for senior students) were significantly associated with frequency of after-school physical activities. After conducting the stepwise process, encouragement from parents (junior students, p < 0.0001; senior students, p < 0.0001) and friends (junior students, p < 0.0001; senior students, p = 0.0001) remained significant across all groups while sibling/cousin support was only significant for junior students (p = 0.0001). As shown in Table 1, these variables remained significant for their respective age groups in the multivariate model once cluster sampling was corrected for.

**Table 1:** Univariate and multivariate analysis of perceived support from various sources for participation in after-school physical activity.

	**Juniors (n = 2,490)**				**Seniors (n = 981)**			
		
	**Total n**	% active*	**Univariate model**	**Multivariate model**^†^	**Total n**	% active*	**Univariate model**	**Multivariate model**^†^
			OR (95% CI)^‡^	OR (95% CI)^‡^			OR (95% CI)^‡^	OR (95% CI)^‡^
**Parent(s)**								
High (2/2 parents)	99	70.0	1.0^§^	1.0^§^	54	72.2	1.0^§^	1.0^§^
High (1/1 parent)	1007	62.6	0.78 (0.47–1.29)	0.87 (0.53–1.44)	281	59.3	0.56 (0.30–1.06)	0.62 (0.33–1.16)
High (1/2 parents)	456	55.5	0.54 (0.43–0.68)	0.64 (0.50–0.80)	166	59.6	0.58 (0.39–0.86)	0.68 (0.45–1.01)
Low (2/2 parents)	753	44.1	0.36 (0.29–0.44)	0.47 (0.38–0.58)	390	42.1	0.33 (0.23–0.48)	0.41 (0.29–0.60)
Low (1/1, no parents)	175	41.1	0.33 (0.24–0.47)	0.46 (0.32–0.65)	90	36.7	0.25 (0.15–0.44)	0.31 (0.18–0.55)

**Sibling/Cousin(s)**								
High	1241	67.9	1.0^§^	1.0^§^	392	65.1	1.0^||^	
Low	1095	46.5	0.45 (0.39–0.53)	0.66 (0.56–0.79)	515	47.0	0.54 (0.41–0.73)	
No sibling	154	46.8	0.51 (0.36–0.71)	0.71 (0.50–1.02)	74	45.9	0.65 (0.39–1.10)	

**Friend(s)**								
High	986	70.1	1.0^§^	1.0^§^	385	70.4	1.0^§^	1.0^§^
Low	1504	48.7	0.45 (0.38–0.54)	0.61 (0.51–0.74)	596	43.6	0.40 (0.29–0.54)	0.49 (0.35–0.69)

**School**								
High	1369	62.3	1.0^§^		546	57.2	1.0^ns^	
Low	1121	50.9	0.67 (0.57–0.79)		435	51.6	0.85 (0.67–1.08)	

*participated in sport, dance, cultural performances, or played active games after school on at least 3 weekdays

† only variable found significant (p <.05) through the stepwise process were included in the final multivariate model

‡ Corrected for cluster effect and controlled for sex and ethnicity

§ p = <.0001; || p = .0003; ns = non-significant

Based on the multivariate models (Table 1) youth who received low parental support were less likely to be considered active after school compared to youth who received high levels of encouragement from both parents. Also, youth who resided in a single parent family but received high support from their sole parent were just as active after school as youth who received high support from two parents. Among junior students with siblings, those who received low support were less likely to be regularly active after-school. Furthermore, low friend support increased the likelihood of not being active after school with reported adjusted OR's of 0.61 (95% CI: 0.51–0.74) and 0.49 (95% CI: 0.35–0.69) for junior and senior students, respectively.

### Lunchtime physical activity

Based on univariate analyzes (Table 2), all sources of support (parents, friends, siblings/cousins, and school) were significantly associated with being active at lunchtime for both junior and senior students. After conducting the stepwise process, only perceived friend support was significantly related to lunchtime physical activity levels for both junior (p < 0.0001) and senior (p < 0.0001) students. Parental support (p = 0.01) and school support (p = 0.03) also remained significant for junior students only. As shown in Table 2, these variables, with the exception of school support, remained significant for their respective age groups in the multivariate model once cluster sampling was corrected for. For both junior (OR: 0.39, 95% CI: 0.32–0.48) and senior students (OR: 0.41, 95% CI: 0.29–0.57), the multivariate models show that students reporting low peer support are less likely to be categorized as active (i.e., mostly played active games) compared to those reporting high levels of encouragement from friends. Also, among junior students from two-parent families, those receiving low support from at least one parent are less likely to be active during lunchtime with noted OR's of 0.68 (95% CI: 0.54 – 0.87) for students with low parental support from two-parent families and 0.77 (95% CI 0.59 – 0.99) for students with high support from only one parent from a dual parent family.

**Table 2:** Univariate and multivariate analysis of perceived support from various sources for participation in lunchtime physical activity.

	**Juniors (n = 2,490)**				**Seniors (n = 981)**			
		
	**Total n**	% active*	**Univariate model**	**Multivariate model**^†^	**Total n**	% active*	**Univariate model**	**Multivariate model**^†^
			OR (95% CI)^‡^	OR (95% CI)^‡^			OR (95% CI)^‡^	OR (95% CI)^‡^
**Parent(s)**								
High (2/2 parents)	99	43.8	1.0^§^	1.0**	54	36.3	1.0^¶^	
High (1/1 parent)	1007	33.3	0.82 (0.50–1.34)	0.92 (0.55–1.54)	281	31.5	0.92 (0.50–1.68)	
High (1/2 parents)	456	33.1	0.64 (0.50–0.82)	0.77 (0.59–0.99)	166	26.5	0.66 (0.44–0.99)	
Low (2/2 parents)	753	26.8	0.51 (0.40–0.65)	0.68 (0.54–0.87)	390	19.7	0.53 (0.37–0.76)	
Low (1/1, no parents)	175	24.6	0.49 (0.33–0.73)	0.70 (0.46–1.04)	90	17.8	0.49 (0.26–0.93)	

**Sibling/Cousin(s)**								
High	1241	43.3	1.0^§^		392	34.4	1.0^||^	
Low	1095	26.7	0.52 (0.43–0.63)		515	21.2	0.60 (0.45–0.79)	
No sibling	154	26.6	0.59 (0.39–0.90)		74	16.2	0.50 (0.21–1.19)	

**Friend(s)**								
High	986	51.8	1.0^§^	1.0^§^	385	39.7	1.0^§^	1.0^§^
Low	1504	23.9	0.34 (0.28–0.42)	0.39 (0.32–0.48)	596	17.3	0.41 (0.29–0.57)	0.41 (0.29–0.57)

**School**								
High	1369	39.2	1.0^§^		546	30.3	1.0^††^	
Low	1121	29.7	0.64 (0.52–0.79)		435	22.7	0.71 (0.51–0.98)	

* played active games most of the time

† only variables found significant (p <.05) through the stepwise process were included in the final multivariate model

‡ Corrected for cluster effect and controlled for sex and ethnicity

§ p = <.0001; || p=.001; ¶ p = .007; ** p = .02; †† p = .04; ns = non-significant

### Active transportation

As shown in Table 3, univariate analyzes identified that only peer support among junior students was significantly associated with frequency of active transportation (i.e., walking/biking at least five times to or from school over a school week). After adjusting for all predictor variables through the stepwise process, friend support (p = 0.005) and school support (p = 0.04) were significant for junior students. No significant variables emerged for senior students, therefore, no multivariate model was tested for this age group. For junior students, the multivariate model, once corrected for cluster sampling, shows that junior students who receive low peer support have a reduced odds (OR: 0.78, 95% CI: 0.66 – 0.92) of undertaking at least 5 trips to or from school by active transportation modes, while low school support was associated with an increased odds of commuting actively on a regular basis (OR: 1.20, 95% CI: 1.02 – 1.40).

**Table 3:** Univariate and multivariate analysis of perceived support from various sources for participation in active transportation to and from school.

	**Juniors (n = 2,490)**				**Seniors (n = 981)**		
		
	**Total n**	% active*	**Univariate model**	**Multivariate model**^†^	**Total n**	% active*	**Univariate model**
			OR (95% CI)^‡^	OR (95% CI)^‡^			OR (95% CI)^‡^
**Parent(s)**							
High (2/2 parents)	99	61.6	1.0^ns^		54	55.5	1.0^ns^
High (1/1 parent)	1007	59.6	0.90 (0.62–1.32)		281	59.3	1.22 (0.67–2.24)
High (1/2 parents)	456	60.7	0.97 (0.79–1.20)		166	53.6	0.96 (0.68–1.37)
Low (2/2 parents)	753	57.0	0.85 (0.71–1.03)		390	49.7	0.93 (0.68–1.28)
Low (1/1, no parents)	175	61.7	1.01 (0.74–1.39)		90	58.9	1.32 (0.77–2.26)

**Sibling/Cousin(s)**							
High	1241	61.3	1.0^ns^		392	57.1	1.0^ns^
Low	1095	58.8	0.91 (0.78–1.07)		515	52.2	0.89 (0.64–1.24)
No sibling	154	57.1	0.87 (0.62–1.22)		74	41.9	0.62 (0.40–0.96)

**Friend(s)**							
High	986	63.4	1.0^||^	1.0^§^	385	55.8	1.0^ns^
Low	1504	57.7	0.81 (0.69–0.95)	0.78 (0.66–0.92)	596	51.8	1.02 (0.77–1.36)

**School**							
High	1369	58.4	1.0^ns^	1.0^¶^	546	51.3	1.0^ns^
Low	1121	61.9	1.14 (0.98–1.33)	1.20 (1.02–1.40)	435	55.1	1.21 (0.91–1.61)

* walked/biked to or from school for at least 5 trips over the last 5 school days

† only variables found significant (p < .05) through the stepwise process were included in the final multivariate model

‡ Corrected for cluster effect and controlled for sex and ethnicity

§ p = .004; || p = .01; ¶ p = .03; ns = non-significant

## Discussion

This study examined the importance of one form of social support (i.e., encouragement to do physical activity) from four support sources (parents, siblings/cousins, friends, and schools) across three time-specific physical activities (after-school physical activity, lunchtime physical activity, and active transportation to and from school). A key and novel finding of this study was that the importance of encouragement from the various sources was dependent on the time-specific activity examined.

### After-school physical activity

In line with prior research [8], we identified that encouragement from parents and friends was a key contributor to youth being active after school irrespective of age cluster. During adolescence, therefore, both parents and friends play an important role in the socialization of teenagers to after school activities. Students who reported receiving high support from at least one parent were just as likely to be active on most days during the after-school period, compared to their peers who received high levels of support from two parents. These findings provide a positive picture that youth from single parent families can be just as active after school as students from two parent families, as long as the available parent provides a high level of encouragement towards his/her offspring. Frequency of after-school activities was also significantly associated with peer encouragement, with students receiving limited support being less likely to be regularly active after school compared to their counterparts receiving higher levels of peer support. In addition, a significant association with sibling/cousin support emerged for junior but not senior students. In particular, among junior students with siblings/cousins, those who received high support were more likely to be active after school. The finding that the importance of sibling/cousin encouragement differed by age lends support to the perspective that during adolescence peers become powerful influencers, more so than family members; a finding similar to those noted in prior research [8,14,16]. This is not unexpected, and is likely a natural consequence of young people increasing their independence from families and expanding social networks external to the family environment as they move through adolescence [28]. Examining the sex, age, number of siblings/cousins and types of activities engaged in after-school may, however, help provide further insight into why this association emerged for junior students only.

### Lunchtime physical activity

In contrast to the findings for after-school activities, friends emerged as the only consistent source of support associated with lunchtime physical activity levels across both age clusters. This is not unexpected as ecological models postulate that influencers most proximal to the target group will have the strongest effect on the desired behavior. During lunchtime and within a school setting the most proximal social force would be a friend, which explains why students who reported receiving low support from their peers were more likely to sit, stand, or walk around during lunchtime rather than play active games. A significant association with parental support emerged for junior students only, with low parental support from two parent families reducing the odds of their offspring to be considered active during lunchtime. For junior students, therefore, as long as they receive high support from either friends or parents, the probability of engaging in active play during lunchtime increases. The lack of an effect of parental encouragement on senior students is potentially indicative of the higher importance of peer influences with increasing age.

### Active transportation

Perceived encouragement to be active was not associated with the regularity of walking or biking to and from school among senior students. For junior students, commuting actively to school was positively associated with friend support but inversely related to school support. The limited findings between perceived encouragement and active transportation is not unexpected as physical environment factors, which have been linked to both active transportation [35] and physical activity in general [36-39], are more likely to impede students undertaking active transport than perceived encouragement. For instance, no matter how much encouragement parents provide to their children to be active, if the family lives too far from school commuting actively is less likely to occur. Considering active transportation occurs outside the home and school environments, examination of social factors at the neighborhood level (e.g., safety, people visible in the neighbourhood, level of neighbour interactions and cohesion) may provide further insight into social influencers on active transportation patterns to and from school.

A particular strength of this study was the large and ethnically inclusive sample. In addition, this study is one of the first to examine the influence of perceived encouragement from various support sources across three time dependent physical activity opportunities that exist within a school day. Also, examining how many parents each participant lived with during a school week allowed the effect of different parental structures (single parent, dual parents, or no parents) to be investigated. The limitations of this study, however, need to be noted. As it was not the purpose of the larger Obesity Prevention in Communities (OPIC) project to obtain a nationally representative sample of youth but rather to over sample for Pacific Island youth with low socioeconomic status, the resulting sample is not representative of the New Zealand youth population. Although the sample composition is similar to that of the New Zealand high school population for gender (approximately 50% females), the sample differed substantially by ethnicity when compared to national statistics. The ethnic composition of the sample was 57% Pacific Island, 20% Māori, 12% European, and 11% Asian and Other compared to 7.6%, 24.5%, 62.4% and 5.0%, respectively, for the New Zealand child population (<15 years old). The generalizability of the findings to the New Zealand youth population must, therefore, be interpreted with some caution. Another limitation is the use of self-report measures [40], an unavoidable limitation as information about participation in specific physical activity contexts (e.g., active transportation) can only be confirmed by this method. Although accelerometers allow examination of physical activity intensity during certain time periods throughout a day, it cannot always distinguish the specific context in which the activity is taking place (e.g., physical activity after school could be due to sports participation or transport related physical activity). Reducing the self-report monitoring period to the week prior to the questionnaire completion day along with recalling frequency of activity during specific time periods, potentially reduce the effect of known associative recall bias of self-reports [40] when compared to longer monitoring frames or when specific duration of physical activities are examined. Although the encouragement questions were based on questions included in prior studies (thereby allowing cross study comparisons), the use of a single question may not accurately capture the complexity of perceived encouragement. Furthermore, school encouragement may have been interpreted differently among participants, in terms of source (e.g., support form head teacher, senior managers/teachers, physical education teachers) and type of encouragement (e.g., supportive school ethos, instrumental support, verbal encouragement). Clarifying the definition of school support is required in future studies. Other limitations include the use of cross-sectional data, which limits the ability to examine the impact of perceived support on the development of physical activity levels during the high school years longitudinally. Also, only one type of perceived support was examined which prevented the impact of overall support on youth activity to be analyzed.

## Conclusion

The findings from this study highlight the importance of proximal social networks on youth activity which should be considered when developing policies and programs looking to promote physical activity among young people. The findings also provide further evidence that parents and friends are the key social influencers of physical activity during adolescence. To determine the true effect of school support on adolescent physical activity, further research is required that utilizes more in-depth and specific question(s) to assess school encouragement and the wider school environment that may impact on perceived encouragement (e.g., the school ethos).

## Competing interests

The author(s) declare that they have no competing interests.

## Authors' contributions

RS is the principal investigator and DS a co-investigator on the Obesity Prevention in Communities (OPIC) project from which these data were obtained. MH, RS, GS and DS were all involved in survey design and acquisition of data. GS provided guidance regarding the statistical analyzes to be applied. GS and GK assisted with the interpretation of the results. MH developed the first draft of the manuscript while all other authors contributed to the writing of the manuscript.
